# White adipose tissue as a target for cadmium toxicity

**DOI:** 10.3389/fphar.2022.1010817

**Published:** 2022-10-06

**Authors:** Sarra Mohammed Attia, Sandra Concepcion Das, Kavitha Varadharajan, Hamda A. Al-Naemi

**Affiliations:** ^1^ Laboratory Animal Research Center, Qatar University, Doha, Qatar; ^2^ Department of Biological and Environmental Science, Qatar University, Doha, Qatar

**Keywords:** cadmium, heavy metals, environmental pollutant, adipose tissue, adipocytes dysfunction, adipokines

## Abstract

Cadmium (Cd) is a widespread heavy metal known as a toxic environmental pollutant. Cd exposure is threatening due to its bioaccumulation trait in living systems that exceeds 35 years without a beneficial biological role. Acute exposure to high Cd doses was reported to impact adipose tissue (AT) function adversely. The main aim of this study is to investigate the effect of low-dose chronic Cd exposure on the genes involved in adipose tissue (AT) functions. Adult male Sprague-Dawley rats were exposed to a low Cd dose (15 mg/kg B.W./day) for 10 weeks. Then, three AT depots-subcutaneous AT (SUB-AT), abdominal AT (AB-AT), and retroperitoneal AT (REtrop-AT) were excised for Cd accumulation measures and gene expression analysis. Adiponectin and leptin gene expression levels were investigated as markers for adipocytes function and homeostasis. Our results showed that Cd accumulated in all the tested adipose depots, but SUB-AT was found to be the depot to most accumulate Cd. Also, it was exhibited that chronic exposure to low Cd doses altered the gene expression of adipocytokines. The levels of adiponectin and leptin mRNA expression were downregulated in all tested AT-depots after Cd exposure. The significant adverse effect on SUB-AT compared to other depots indicates different responses based on AT depots location toward Cd exposure. Collectively, these results suggest a toxic effect of Cd that influenced adipocyte function.

## Introduction

Environmental pollution is increasing due to the extensive urbanization and expanded industries. Cadmium (Cd) is classified as a toxic environmental pollutant that is listed among the top ten toxic substances by the Agency for Toxic Substances and Disease Registry (ATSDR, 2019). The recent global production of Cd reached 23,000 metric tons, highlighting the current challenge of Cd exposure worldwide (USGS, 2020). The main routes of Cd exposure are inhalation and ingestion of contaminated food and water (Andjelkovic et al., 2019; Fatima et al., 2019). Cadmium pose a toxic risk because of its prolonged biological half-life (10–30 years) with no known beneficial physiological function (Jacobo-Estrada et al., 2017; Rahimzadeh et al., 2017). Cadmium accumulates in different tissues such as liver, kidney, skeletal muscle, pancreas, and also the adipose tissue (Ficková et al., 2003; Lee et al., 2012; Buha et al., 2018).

Adipose tissue (AT) is a key organ that regulates various physiological processes such as lipid metabolism and energy homeostasis. In addition, AT is responsible for releasing factors known as adipokines that regulate appetite, energy expenditure, and fat distribution. Adipokines are involved in metabolic regulation and play an essential role in maintaining systemic functions such as inflammatory and immunological responses, vascular events, reproductive functions, appetite regulation, and insulin sensitivity (Henriques et al., 2019). Additionally, some of these secreted adipokines exert both autocrine and paracrine actions, which mainly affect the processes of AT remodeling, angiogenesis, and adipogenesis (Ordovas and Corella, 2008; Henriques et al., 2019; Attia et al., 2022). The AT secretory status depends on the changes of cellular tissue composition, including alterations in the phenotypes, numbers, and site of adipose tissue depots (Ouchi et al., 2011). There are two types of adipokines- pro-inflammatory such as leptin, monocyte chemoattractant protein 1 (MCP-1), tumor necrosis factor-α (TNF-α), interleukin 6 (IL-6) and anti-inflammatory such as adiponectin and interleukin 10 (IL-10) (Mancuso, 2016; Hui and Feng, 2018; Henriques et al., 2019).

Regarding the adipokines, leptin is an adipokine secreted by AT and was the main reason for adipose tissue recognition as an endocrine organ when first discovered in 1994 (Zhang et al., 1994). Leptin signals have an essential contribution to regulating AT metabolism, appetite, satiety, puberty, fertility, and reproductive function (Fasshauer and Blüher, 2015; Stern et al., 2016). Moreover, leptin can directly increase pro-inflammatory cytokines such as TNF-α and IL-6 in monocytes and enhance the production of chemokines like MCP-1 and IL-8 in macrophages and the lipid mediators PGE2 cysteinyl leukotrienes (Ouchi et al., 2011; Mancuso, 2016). Adiponectin is an adipokine produced exclusively by adipocytes with a high level in the blood that ranges between 3 and 30 μg/ml, and it targets different cell types (Fasshauer and Blüher, 2015; Mancuso, 2016). The metabolic properties of adiponectin are favorable since it is an anti-inflammatory adipokine that can inhibit the activation of nuclear factor kappa-light-chain-enhancer of activated B cells (NF-κB). As a result, it inhibits inflammation, reduces the expression of TNF-α and IL-6, and regulates glucose metabolism and energy homeostasis (Chandrasekar et al., 2008; Novotny et al., 2012; Vasiliauskaité-brooks et al., 2017). Studies showed that a dysregulation in the adipocyte secretion of adiponectin, leptin, resistin, and TNF-α is related to increased risk of type 2 diabetes and arteriosclerosis (Kawakami et al., 2010; Samaras et al., 2010). The crosstalk between the adipokines and cytokines under cadmium exposure were extensively reviewed by Attia et al. (2022).

White adipose tissue (WAT) is considered the main site of metabolic dysregulation in several metabolic diseases (Henriques et al., 2019). There are two main subtypes of WAT: the subcutaneous and the visceral. The latter also can be further subdivided into omental, mesenteric, perirenal, and peritoneal fat depots (Choe et al., 2016). Subcutaneous AT is located in the innermost layers of the skin and has a primary function of energy storage (Badimon and Cubedo, 2017; Henriques et al., 2019; Kahn et al., 2019). Moreover, subcutaneous AT is responsible for thermal insulation and providing a protective cushion against mechanical damage (Choe et al., 2016; Chait and den Hartigh, 2020). On the other hand, the visceral AT is located in the internal organs and is known for its high metabolic response (Badimon and Cubedo, 2017; Kahn et al., 2019). Both subcutaneous AT and visceral AT have different metabolic functions and different adipokine expression profiles.

Adipose tissue is a potential target for Cd accumulation. Recent study reported that Cd accumulates in AT of the human body with an average concentration of 12.6 µg/kg (Egger et al., 2019). Moreover, results reported by Echeverría et al. (2019) showed that the mean Cd concentration in AT of breast and waist regions was 32 and 42 µg/kg, respectively. The authors correlated this accumulation of Cd in AT with several parameters such as age, smoking, the types of food consumed, and body mass index. Data collected earlier from animal studies showed similar results. Kawakami et al. (2010) reported a correlation between Cd dose increments and elevation of Cd concentration in AT of male SIc: ICR mice. The risk of Cd accumulation in AT includes disrupting its capability to accommodate the surplus energy and produce the required adipokines for its endocrine function. Consequently, this may affect systemic homeostasis since AT occupies a large part of the whole body. Of note, few studies investigated the direct effect of Cd on AT function. Thus, this study aims to investigate the effect of chronic exposure to low dose of Cd on AT, where the pattern of adipocyte secretion will be evaluated to assess the white adipose tissue function.

## Materials and methods

**FIGURE 1:**
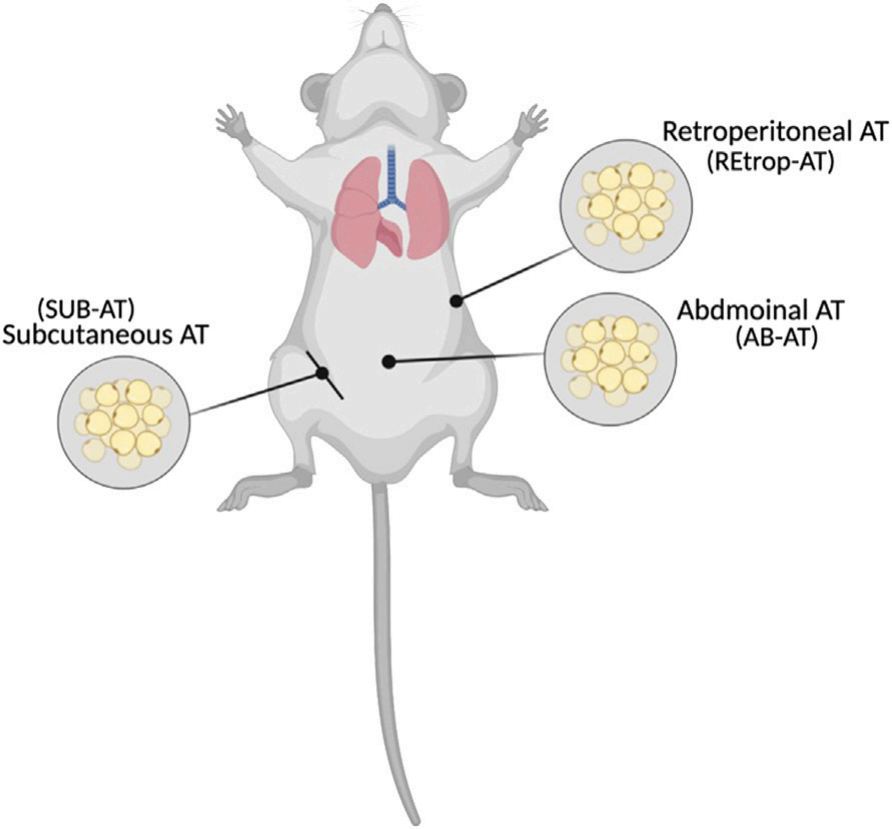
Locations of the collected adipose tissues (Created with BioRender.com).

The study design was established by Al-Naemi and Das (2020), and approved by the Institutional Animal Care and Use Committee (Approval# QU-IACUC 038/2017) Briefly, adult male Sprague-Dawley (SD) rats (8 weeks old) were divided into two groups, control (C) and cadmium-treated (Cd-T). The control group received standard chow and normal drinking water while cadmium treated group received standard chow and cadmium in drinking water with dose of 15 mg Cd/kg body weight as CdCl_2_ (BDH Chemicals, England) for 10 weeks, *ad libitum*. During the study, the health status of the animals were observed and recorded. After 10 weeks, the animals were anesthetized, sacrificed and adipose tissue depots: subcutaneous and visceral (abdominal, and retroperitoneal) were collected as indicated in Figure 1, frozen in liquid nitrogen and stored in the repository at −80°C. The administered dose was chosen to represent the human equivalent dose (HED) of 2.4 mg/kg and similar to the reported intake in epidemiological studies (Reagan-Shaw et al., 2008, Bernhoft, 2013; Chunhabundit, 2016; Ghosh et al., 2018).

### Cadmium accumulation in adipose tissues

Sample preparation was adapted as previously published by Ishak et al. (2015). Harvested adipose tissue samples were weighed and digested in 69% of trace metal analysis grade nitric acid (VWR International) overnight at room temperature. Following digestion, samples were incubated at 60°C for 1 h, allowed to cool, and incubated in hydrogen peroxide at 60°C for 1 h. Then, samples were diluted to a final volume of 10 ml with deionized water and filtered using a 0.2 µm injection filter (GE Healthcare Life Sciences, United Kingdom) to remove any debris. Digested samples were analysed for cadmium quantification by Inductively Coupled Plasma Optical Emission Spectrometry (ICP-OES, Model: Optima 7300 DV) performed by Central Laboratories Unit, Qatar University. The limit of detection for the instrument is 0.0001 µg/g. Concentrations are represented as µg/g of tissue.

### Evaluation of Cd effect on white adipose tissues

The effect of Cd on WAT was evaluated by gene expression assay. Adipose tissue previously stored at −80°C were homogenized using both liquid nitrogen and probe sonicator (QSonica 500), then total RNA was extracted from adipose tissue using TRIzol™ LS Reagent (ThermoFisher Scientific, United States; 10296010). Slight modifications were followed at the washing step, where the RNA pellet was resuspended in ice cold 75% ethanol and kept at −20°C overnight. Then, the sample was washed three times to enhance the RNA purity. Total RNA was quantified using nanophototmeter (Implen; P330). A known amount of RNA (150 ng) was reverse transcribed into cDNA using the high capacity cDNA transcription kit (Applied Biosystems, Lithuania) following the manufacturer’s instructions. Final volume of the reaction was 20 µl and stored at −20°C until the performance of Real Time-PCR gene expression assays. RT-PCR was performed using diluted cDNA (1:3) and TaqMan^®^ Fast Advanced Master Mix (Applied Biosystems, United States) for six targets as following: adiponectin (Rn00595250_m1), leptin (Rn00565158_m1), MCP-1 (Rn00580555_m1), IL-6 (Rn01410330_m1), IL-10 (Rn01483988_g1), TNF-α (Rn01525859_g1), GAPDH (Rn01775763_g1). GAPDH was assigned as the endogenous control gene. The amplification was carried out in QuantStudio 6 flex system (Applied biosystem™). The relative quantity of gene expression was calculated using 2^−∆∆Ct^ method. Results are presented as fold change (log2) versus the mean values of the control samples normalized against the endogenous gene.

### Statistical analysis

Gene expression results were generated using 2^−∆∆Ct^ method. Experiments were conducted in duplicate. The values are presented as means ± SEM. Data was analyzed by Kruskal-Wallis test, one-way and two-way ANOVA followed by Tukey’s multiple comparison test. Statistical analysis was performed using GraphPad Prism version 9.3 (GraphPad Software, San Diego, California United States, www.graphpad.com). *p*-value < 0.05 is considered a significant value.

## Results

### Effect of Cd exposure on the monitored parameters

Body weight, water, and chow intake of control and Cd-T rats were recorded weekly for 10 weeks (Table 1). Initially, no significant differences were observed in the body weight. However, at week 10 statistically significant difference was observed in the mean body weight between the groups (*p*-value < 0.001). The weekly water intake in both groups remained stable with slight non-significant fluctuations during the study period, with a significantly reduced intake in the Cd-T group (*p*-value < 0.001). The Cd-T group had a reduced initial water intake of about 52% relative to the control. At the end of the study, the difference in water intake of the Cd-T group was 11 percent lesser than the control. The initial mean chow intake of the Cd-T group was about 15% lesser than the control group, whereas the final mean chow intake of the Cd-T group was found to be like the control group. The differences in weekly chow intake were found to be statistically significant from week 1 until week 10 (*p*-value < 0.05).

**TABLE 1:** Body weight, water intake and chow intake during the period of the study.

	Control group		Cd-treated group	
	Initial (Week 1)	Final (Week 10)	Initial (Week 1)	Final (Week 10)
Mean body weight (*g*)	378.06 ± 4.63	579.32 ± 7.87	358.88 ± 5.04	512.34 ± 10.58***
Mean water intake (*mL/rat/week*)	276.68 ± 16.47	227.67 ± 30.54	132.78 ± 1.42***	203.12 ± 39.86***
Mean chow intake (*g/rat/week*)	185.45 ± 2.88	182.65 ± 6.59	158.11 ± 2.66*	180.68 ± 22.21*

*p < 0.05, ***p < 0.001 when compared to control group at the corresponding time point.

### Accumulation of Cd in adipose depots

The concentration of Cd in the adipose depots was determined by Inductively Coupled Plasma-Optical Emission Spectrometry (ICP-OES). The three adipose depots collected from Cd-T rats exhibited Cd accumulation (Table 2). The increasing order of Cd accumulation was found to be in REtrop-AT < AB-AT < SUB-AT. The differences in the mean Cd concentrations, considering each depot, was found to be highly significant in SUB-AT (*p*-value < 0.01) compared to AB-AT and REtrop-AT.

### Evaluation of Cd exposure on adipose tissues

**FIGURE 2:**
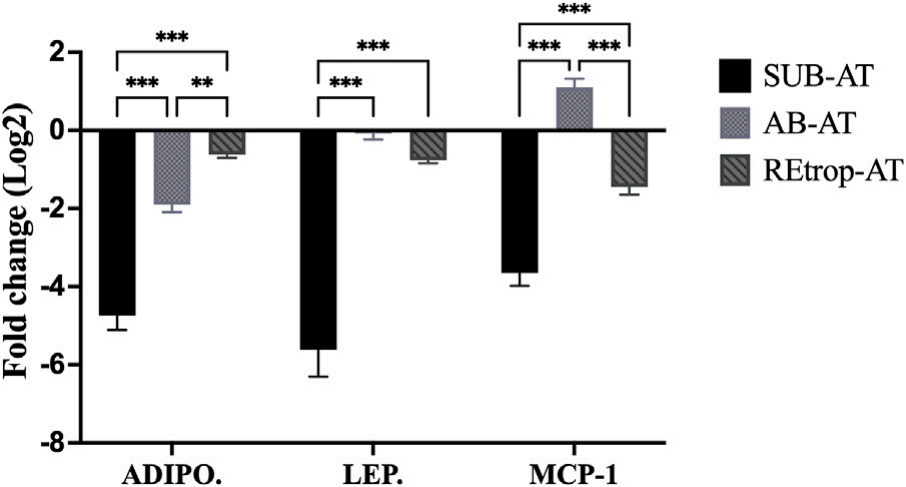
Effect of Cd treatment on the expression level of adiponectin (ADIPO.), leptin (LEP.) and monocyte chemoattractant protein 1 (MCP-1) in three different adipose depots of male Sprague-Dawley rats after 10 weeks Cd treatment. Gene expression results were generated using 2^−∆∆Ct^ method and the mean fold change (log2) values of the targets mRNA expression were normalized to control samples and the endogenous gene. (significance: more than 2-fold change) and Two-way ANOVA was performed using GraphPad Prism version 9. The significant difference is represented by **p < 0.01, ***p < 0.001, (*n* = 6).

Adipose tissue samples from control and Cd-T rats were analyzed for gene expression to explore the effect of Cd on AT. In Figure 2, the expression patterns of adipokines (adiponectin and leptin) and chemokine (MCP-1) are illustrated in the three fat depots. Subcutaneous adipose tissue exhibited a pattern of downregulated expression for both adiponectin and leptin. Similarly, adiponectin and leptin gene expression were decreased in REtrop-AT and AB-AT. However, the fold change for both adiponectin and leptin in SUB-AT was significant by more than two folds compared to REtrop-AT and AB-AT. For chemokine MCP-1, a downregulation trend is observed in both REtrop-AT and SUB-AT with a significant value (−3.8-fold) in the latter. Conversely, a trend of up-regulation of MCP-1 was observed in AB-AT.

### Cd exposure and the inflammatory mediators in adipose tissue

**FIGURE 3:**
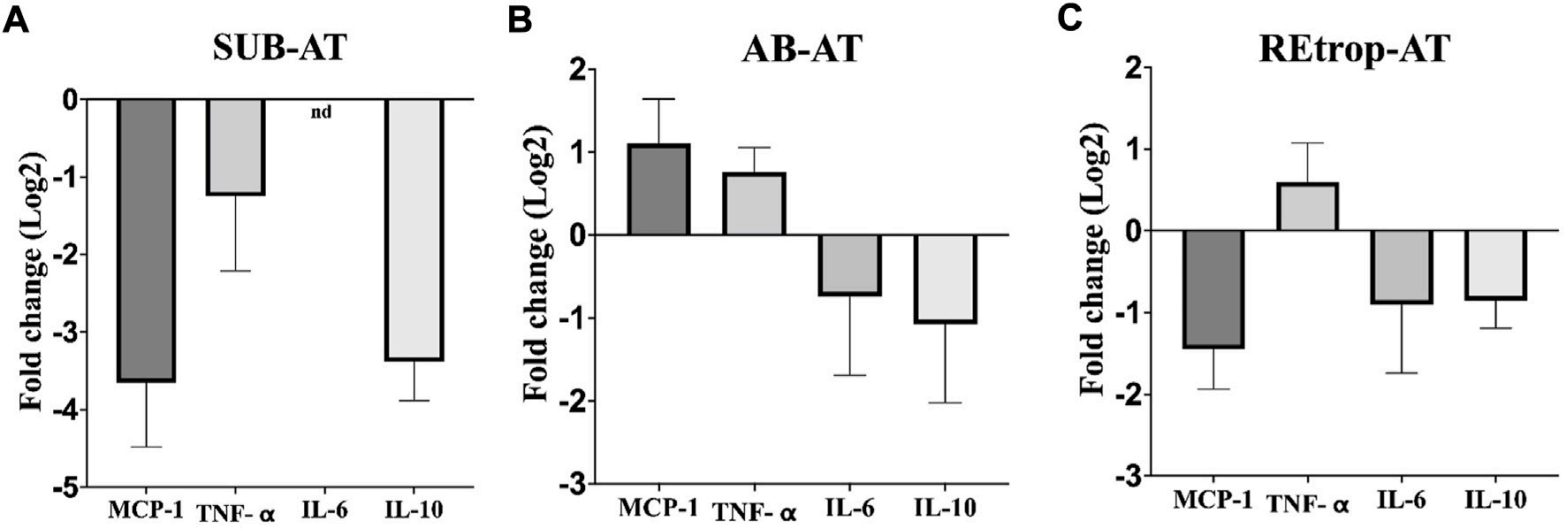
The expression level of MCP-1and the inflammatory mediators in three different adipose depots of male Sprague-Dawley rats. **(A)** Subcutaneous adipose tissue (SUB-AT), **(B)** Abdominal adipose tissue (AB-AT), **(C)** Retroperitoneal adipose tissue (REtrop-AT). Gene expression results were generated using 2^−∆∆Ct^ method. The mean fold change (log2) values of the targets mRNA expression were normalized to control samples and the endogenous gene. significance: more than 2 fold change, compared to the control. (n = 6). nd, not detected.

**FIGURE 4:**
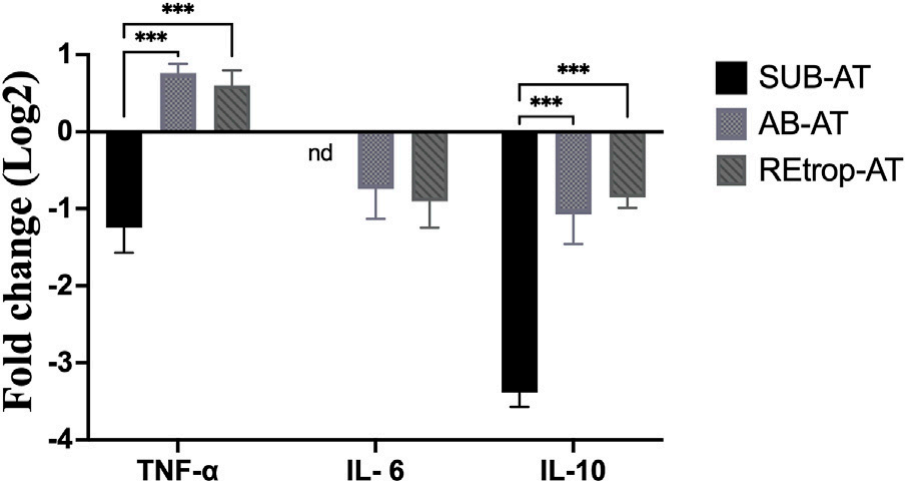
The comparison between the three adipose depots in term of the expression level of the inflammatory mediators. (black) Subcutaneous adipose tissue (SUB-AT), (dotted) Abdominal adipose tissue (AB-AT), (diagonal lined) Retroperitoneal adipose tissue (REtrop-AT). Gene expression results were generated using 2^−∆∆Ct^ method and the mean fold change (log2) values of the targets mRNA expression were normalized to control samples and the endogenous gene. Two-way ANOVA and Tukey’s post hoc test was performed using GraphPad Prism version 9, (*n* = 6). ***p < 0.001. nd, not detected.

After observing differences in MCP-1 among the depots, we investigated the relationship between the expression level of MCP-1 and the inflammatory mediators in each depot under the condition of Cd exposure (Figure 3). Results showed an upregulation trend of TNF-α in AB-AT with no significant fold change. Similar results were observed in REtrop-AT. In contrast, in SUB-AT, TNF-α expression level showed a downregulation trend with no significant fold change. Both IL-6 and IL-10 were downregulated in all AT depots despite varying levels of change of MCP-1. Also, IL-6 was below the detection limit in SUB-AT. Put together, within each AT depot, there was no significant difference between the expression level of MCP-1 and the inflammatory mediators (TNF-α, IL-6, and IL-10). However, when comparing the effect of Cd exposure on the inflammatory mediators between depots (Figure 4), a significant difference was found between SUB-AT and other depots for TNF-α and IL-10 with a *p*-value < 0.001.

## Discussion

The accumulative properties of Cd in living systems were found to impose adverse effects on tissues and influence their functions (Kumar and Sharma, 2019; Genchi et al., 2020). In this study, the effect of Cd on adipose tissue was investigated in three different depots of adult male SD rats. Our results showed that Cd exposure caused a significant decrease in rats’ body weight. The reduction of the body weight agrees with former studies that recorded Cd exposure caused a downregulation in the body weight of murine (Kawakami et al., 2010; Prabhu et al., 2020). The reduction of the body weight in the Cd-T group is suggestive of either losing adipocytes or dysregulated adipogenesis. Additionally, our results showed a downregulation of adiponectin and leptin mRNA expression levels in all AT depots. This downregulation of adiponectin and leptin mRNA expression level agrees with a previous finding, reporting that acute Cd exposure significantly decreased the mRNA expression level of adiponectin in mice (Kawakami et al., 2010). Another study conducted by Kawakami et al. (2013) using metallothionein-null mice reported that Cd exposure reduced the expression level of leptin and adiponectin in a dose-dependent manner. In our experimental model, we administered chronic low dose exposure of Cd which resulted in a decline in body weight and the expression level of adiponectin and leptin in Cd-T SD rats. The added value of the current work comes from the experimental design that was established to be realistic and proportional to the population exposure in real life (Das et al., 2021).

Under physiological conditions, the mature differentiated adipocytes produce adiponectin and leptin. Therefore, changes in the production of these adipokines are used as markers to assess changes in adipocytes maturation and functions. Conventionally, the expression patterns of adipokines differ between adipose depots (Lee et al., 2013). Subcutaneous adipose tissue expresses leptin and adiponectin more than visceral adipose tissue (Samaras et al., 2010; Lee et al., 2013; Mazaki-Tovi et al., 2016). This aligns with our results where the expression level of adiponectin and leptin is greater in SUB-AT than AB-AT and REtrop-AT.

However, Cd exposure caused a decrease in the gene expression of adiponectin and leptin and was significantly downregulated by more than two folds in SUB-AT (Figure 2). These changes in the mRNA expression pattern of adiponectin and leptin can be linked with the disruption of adipogenesis including adipocytes maturation. Reaching the maturation stage and achieving the healthy expansion for adipocytes are regulated by critical factors such as peroxisome proliferator-activator receptor gamma (PPARγ) and CCAAT/enhancer-binding protein alpha (C/EBPα). A previous study reported that Cd adversely affects the differentiation of preadipocytes by downregulating the expression level of PPARγ and C/EBPα in 3T3-L1 adipocytes (Lee et al., 2012). This also accords with studies by Kawakami et al. (2010) and Kawakami et al. (2013) where results showed that Cd exposure altered the expression level of the critical regulators of adipogenic differentiation, PPARγ and C/EBPα in mice models. Alterations of adiponectin and leptin expression levels indicate the abnormal adipocytes which could be attributed to disrupted maturation process.

The level of Cd accumulation differs in the three adipose depots. The Cd accumulation results (Table 2) showed that the SUB-AT has the highest amount of Cd among other depots, elucidating its vital role as a sink for storage and buffering of Cd. These accumulated Cd levels resulted in a significant downregulation of adiponectin, leptin and MCP-1 expression levels in SUB-AT compared to other depots (Figure 2). According to literature, MCP-1 production and the macrophages’ infiltration are enhanced by leptin which is a pro-inflammatory adipokine (Mancuso, 2016). However, in the current work, leptin is downregulated, which could explain the downregulated trend of MCP-1. Moreover, leptin and MCP-1 expression levels are correlated with adiposity (Bruun et al., 2005; De Victoria et al., 2009). Thus, the reduced body weight could negatively affect their expression level. A prior study investigated the inflammatory infiltration patterns in different AT depots and reported that visceral AT had more macrophages than subcutaneous AT (Jonas et al., 2015). Moreover, visceral AT is associated with inflammatory events. This could explain the upregulated trend of MCP-1 and TNF-α in AB-AT of our study (Figure 3).

**TABLE 2:** Accumulation of cadmium in the different white adipose depots.

Cd concentration (µg/g tissue wt.)	Cadmium-treated group (*n* = 8)
Subcutaneous adipose tissue	2.285 ± 0.600**
Abdominal adipose tissue	0.255 ± 0.070
Retroperitoneal adipose tissue	0.189 ± 0.097

Concentration is represented as mean ± S.E.M. Data was analyzed using Kruskal-Wallis test. **: *p* < 0.01 when compared between the adipose depots.

The link between the abnormal adipocytes and the induction of inflammation, such as in the case of obesity, is extensively reported (Rull et al., 2010; Greevenbroek et al., 2016). This link is characterized by the presence of activated macrophages and inflammatory cytokines such as TNF-α and IL-6. These cytokines mediate the inflammatory response and are produced by macrophages. Moreover, MCP-1 is responsible for the recruitment of monocytes/macrophages and the induction of inflammatory cytokines in the states of AT abnormality. Therefore, inflammatory cytokines can be used as markers for the activity of macrophages. Additionally, macrophages’ function is determined by macrophages’ quantity, activation state, and metabolic phenotypes (Li et al., 2020). Under pathological conditions, adipose tissue macrophages (ATM) can exhibit mixed phenotypes in response to the local environment (Wang et al., 2021). Furthermore, studies found that preadipocytes have the ability to differentiate into macrophages which highlights the plasticity of AT under different conditions (Charrière et al., 2003; Thomas and Apovian, 2017). Based on the literature, Cd adversely affects the differentiation capacity into macrophages and interferes with the immune cells’ development (Wang et al., 2021). As mentioned earlier, Cd can disrupt the differentiation of preadipocytes through diminishing the transcription factor PPARγ. Another vital role of PPARγ is promoting the differentiation of macrophages into the M2 phenotype (Thomas and Apovian, 2017). Adipose tissue-derived MCP-1 was found to be associated with resident macrophages content, stromal vascular cells, and AT location under both conditions (Bruun et al., 2005). Our results show a trend of downregulation of both IL-6 and IL-10 in all AT depots (Figure 4). However, TNF-α showed a trend of upregulation in AB-AT and REtrop-AT but not in SUB-AT (Figure 4). Thus, the downregulation of both anti-inflammatory and pro-inflammatory mediators further supports the hypothesis of impaired macrophages. Likewise, the impaired differentiation capacity of preadipocytes could explain the downregulation of MCP-1 and the inflammatory cytokines.

According to the literature, former studies showed that Cd significantly reduced the phagocytic activity and decreased the inflammatory responses of murine macrophages in a dose-dependent manner (Loose et al., 1978; Jin et al., 2016). Moreover, a subtoxic Cd dose (10 µM) was found to inhibit the expression level of both IL-10 and IL-6 in murine macrophages (Riemschneider et al., 2015). Furthermore, Cox et al. (2016) proposed that Cd can induce immune dysfunction in macrophages and confirmed it with the lipopolysaccharide treatment after Cd exposure to find that macrophages’ transcription and cytokine release abilities were disrupted (Cox et al., 2016). A single-cell transcriptomic study reported that chronic Cd exposure induces phenotypic alterations in the immune system and reduces the number of monocytes when comparing the circulating immune cells with the plasma Cd level (Lu et al., 2021). Taken together with the current work, chronic Cd exposure disrupts the function of immune cells, especially macrophages.

Former studies that investigated the macrophages infiltration patterns in different AT depots reported that in normal-weight and obese conditions, visceral AT was found to have more macrophages than subcutaneous AT (Jonas et al., 2015). Subcutaneous AT acts as a metabolic sink that stores excess free fatty acids and glycerol in the form of triglycerides. Visceral AT accumulates when the capacity of SUB-AT is exceeded due to chronic stress (Ibrahim, 2010). Also, visceral AT is associated with inflammatory events. This could explain the upregulation trend of MCP-1 and TNF-α in AB-AT of our study. Although the MCP-1 expression level was found to be disrupted, the inflammatory markers were found to be downregulated which suggests that chronic exposure to low-dose Cd is not an inflammatory promotor. It is possible that Cd exposure negatively affected SUB-AT but not to the level that causes significant lipid accumulation in visceral AT.

On the other hand, Kawakami et al. (2013) reported an increment in the number of macrophages and elevation of the mRNA expression level of MCP-1 in the MT-null mice model in a dose-dependent manner. A recent study reported that acute exposure of Cd has a pro-inflammatory effect on human adipocytes (Gasser et al., 2022). An additional study reported acute Cd exposure caused an upregulation of MCP-1 mRNA expression levels in glioblastoma cell lines (Kasemsuk et al., 2020). Moreover, chronic low dose exposure to Cd was found to induce inflammatory infiltration in hepatocytes with an upregulation of MCP-1 mRNA expression level in the pubertal mice model (Li et al., 2021). However, as mentioned earlier, our study followed a different experimental design, including different conditions, tissues, and models which explain the different outcomes regarding MCP-1. This was evident in an *in vitro* study comparing mouse and rat macrophage cell lines which reported that mouse macrophage cell lines were more sensitive to Cd exposure than rat macrophages cell lines (García-Mendoza et al., 2019). Despite the dose, acute exposure of Cd caused an upregulation of MCP-1 mRNA expression level within the first 24h, but the longer time of Cd exposure caused downregulation of MCP-1 expression level for two rats strains (Harstad, 2002). This suggests a different response based on the duration of Cd exposure. Thus, the downregulation of MCP-1 after 10 weeks of exposure to low Cd dose in the current study is the response of ATs of the SD model to the chronic Cd exposure treatment. Of note, the expression of TNF-α was not significant, which may indicate that low-dose Cd is not a pro-inflammatory factor in AT. This is in accordance with previous studies reporting that the pro-inflammatory cytokines were downregulated in the condition of low-dose Cd (Låg et al., 2010; Messner and Bernhard, 2010).

## Conclusion

In conclusion, the current work reported that chronic low-dose Cd exposure leads to accumulation of cadmium in AT depots and the most accumulated amounts were detected in SUB-AT. This altered the gene expression profile of adiponectin and leptin which resulted in its downregulation. The dysregulation of these vital adipokines indicates a toxic effect of Cd that influences adipocyte functions. To the best of our knowledge, the present study is the first to investigate the effect of chronic low-dose Cd on different white adipose depots. Further investigation is required to study the effect of chronic low-dose Cd on the protein level to explore whether Cd induces posttranscriptional alterations that change the functionality of AT proteins. In addition, a histopathological study is required to study the structural changes of each depot. The outcomes of these experiments could further elucidate the role that Cd plays through adipocytes in developing various metabolic complications.

## Data Availability

The original contributions presented in the study are included in the article/supplementary materials, further inquiries can be directed to the corresponding author.
